# Different Categories of Social Media Use and Their Association With Body Image Among Adolescents in 42 Countries

**DOI:** 10.3389/ijph.2024.1606944

**Published:** 2024-07-03

**Authors:** Meyran Boniel-Nissim, Michela Bersia, Natale Canale, Henri Lahti, Kristiina Ojala, Oya Ercan, Anna Dzielska, Joanna Inchley, Paola Dalmasso

**Affiliations:** ^1^ Department of Educational Counseling, The Max Stern Academic College of Emek Yezreel, Emek Yezreel, Israel; ^2^ Department of Public Health and Pediatrics, University of Torino, Turin, Italy; ^3^ Department of Developmental and Social Psychology, University of Padova, Padua, Italy; ^4^ Faculty of Sport and Health Sciences, University of Jyväskylä, Jyväskylä, Finland; ^5^ Research Centre for Health Promotion, Faculty of Sport and Health Sciences, University of Jyväskylä, Jyväskylä, Finland; ^6^ Istanbul University-Cerrahpasa,Cerrahpasa Medical Faculty, Istanbul, Türkiye; ^7^ Department of Child and Adolescent Health, Institute of Mother and Child, Warsaw, Poland; ^8^ MRC/CSO Social and Public Health Sciences Unit, University of Glasgow, Glasgow, United Kingdom

**Keywords:** social media use, HBSC, adolescence, body image, body weight congruence

## Abstract

**Objectives:**

Social media has become integrated into adolescents’ lives and influences body image perceptions. Our study examined four patterns of social media use (SMU): non-active, active, intensive, and problematic. We hypothesised that intensive SMU and problematic SMU would be associated with negative body image (negative subjective body weight) and over/underestimated body weight congruence, compared to non-active and active SMU. In addition, we expect these associations to be stronger for girls.

**Methods:**

Data from 190,892 respondents aged 11, 13, and 15 from 42 countries involved in the Health Behaviour in School-aged Children study were analysed.

**Results:**

Findings revealed higher rates of intensive or problematic SMU among adolescents who perceived themselves as too fat or too thin. Two-level regression analyses showed intensive and problematic SMU as more likely to perceive themselves as too fat or too thin than active users. The association was significant among intensive and problematic girl social media users, whereas, among boys, the relationship was only significant for problematic users.

**Conclusion:**

Our findings highlight the importance of assessing SMU patterns to evaluate associations with body image.

## Introduction

Adolescents rely heavily on electronic media, mainly social media (Twenge et al., 2019). According to the PEW Research Centre of American teenagers, TikTok, Instagram, and Snapchat were the most used by 59%–67% of those aged 13–17 [1]. The Global Kids Online project explored children’s internet use across various European countries and highlighted that around two-thirds of 9–17-year-olds in Europe reported using social media [2]. Additionally, the Health Behaviour in School-aged Children (HBSC) survey conducted in 2017/18 encompassed 45 European and North American countries and regions indicated that approximately one-third of adolescents use social media almost constantly throughout the day to communicate with their peers [3].

The frequent use of social media has raised concerns about the possible implications on the mental health of adolescents [4]. However, the *digital Goldilocks hypothesis* [5] states that, up to a certain point, adolescents’ wellbeing increases as their media use increases, whereas, after that point, it is associated with a decrease in wellbeing. Przybylski and Weinstein [5] revealed that the relationship between media use and mental wellbeing was not linear but curvilinear, whereby moderate involvement was the optimal condition.

Previous studies have supported the *digital Goldilocks hypothesis* [5] using four categories of SMU concerning adolescents’ substance use [6], social relations [6] and sleep patterns [7]. The four categories distinguish between patterns of SMU: non-active use, active use, intensive use, and problematic use. For instance, active social media users contact online with others daily without any sign of addictive-like symptoms. Intensive social media users present with frequent usage that can point to unbalanced use, however, without the presence of addictive-like behaviour. While problematic SMU involves a loss of control and addiction-like symptoms impair daily functioning [6, 8].

In the current study, we suggest using the four categories and their associations with another essential aspect of adolescents’ lives: body image.

### Body Image and Social Media Use

Body image is a psychological construct involving body-related thoughts, beliefs, emotions, and behaviours [9]. Negative body image is defined as a person’s negative thoughts and feelings about their body based on their subjective body weight perception [10]. Individuals suffering from poor body image often experience dissatisfaction with their weight and/or body shape [11]. Body weight congruence (BWC) refers to the discrepancy between actual and perceived body size. Inadequate weight perception, especially weight overestimation, is recognised as a critical component of negative body image and is a known factor in the development of eating disorders [12]. Perceiving one’s body weight as “too slim” or “too fat” was associated with poorer mental wellbeing, regardless of weight status [13, 14].

Literature provides a theoretical framework to present the vital role of social media in body image [15]. The *Sociocultural Theory* has emerged as one of the primary frameworks for conceptualising body image. This theory posits that social agents such as the media, peers, and parents convey strong messages regarding the importance of appearance and pressure to conform to unrealistic body ideals [16–18]. Social media combines aspects of both media and peer influences, as it provides a vehicle for images portraying appearance ideals and an interactive medium allowing for peer feedback through applications such as WhatsApp, TickTok and Instagram [19, 20].

Body image may thus happen directly or indirectly via mediating mechanisms such as internalising body ideals and appearance comparisons. Internalisation means accepting and following body ideals as personal goals [21], whereas appearance comparisons involve evaluating one’s appearance relative to others [22]. Popular adolescent social media platforms, such as Instagram and Snapchat, contain a plethora of idealised body-related content, which tends to endorse muscular ideals (characterised by a v-shaped torso, visible abs, large biceps) and lean/athletic ideals (represented by a toned body with low body fat) for men and boys. For women and girls, social media content is more likely to endorse slim ideals (defined by a lean physique with low body fat and a tight waist), fit/athletic ideals (characterised by a lean and muscular physique), and curvaceous ideals (represented by a thin waist and big bosom/bottom) [23].

Social media platforms may negatively impact adolescent body image by emphasising the significance of looks and unrealistic body ideals [24, 25]. Previous studies have found that adolescents approve and strive for body ideals despite recognising their unrealistic nature [26, 27] and that greater levels of self-objectifying SMU predict body shame among adolescents [28].

Studies linking the use of social media and body image refer mainly to exposure to images and uploading selfies (e.g., [28]). However, today’s youth’s communication on social media includes sharing photos, referring to videos and pictures of their peers or others, commenting on a published story, etc. In other words, exposure to visual content exists within the routine communication of adolescents due to the large amount of documentation they produce and their reference to the documentation of their peers and others. Research showed that social media interaction significantly affects high school students’ self-presentation [29]. Adolescents with large media networks have increasing demands for relationship maintenance, which results in increased photo sharing [30].

Taking and posting selfies on social media is one of the most popular activities associated with adolescents’ SMU, representing a valuable tool to increase their self-presentation via others’ approval. The pictures can be posted through private or group conversations on social media (such as WhatsApp) and/or the private profile or story. Higher exposure to visual content on social media might lead to more social comparisons and appearance concerns reinforcement, possibly creating a vicious cycle; body image-based digital activities may lead to dissatisfied individuals with their appearance to create and manage their best online self-presentation, leading to potentially excessive or problematic SMU, that will maintain their appearance concerns [31].

Although girls usually spend more time on social media (e.g., Instagram and Snapchat) than boys [32], both boys and girls report concerns about appearance pressures and negative evaluations on social media [33]. Gender-dependent differences seem to exist as qualitative research reveals adolescent girls to be less able to control media messages and more often identify social media as the trigger for their body dissatisfaction [34]. In girls aged 11–15, time spent on social media was significantly related to the internalisation of the thin ideal, body surveillance, drive for thinness and more self-objectification [28, 35]. In addition, girls are significantly more involved in and impacted by feedback indices such as “likes” and “comments” [36]. However, much cross-sectional research on social media use and body image focuses on female samples (e.g., [37]) to the exclusion of adolescent boys [38].

### The Current Study

Given adolescents’ widespread use of social media and the importance of body image to their development, mental health, and wellbeing, it is necessary to increase the comprehension of the association between SMU categories and body image to mitigate negative impacts. Despite emerging studies linking SMU to body image (e.g., [39–41]), the evidence is still sparse. While previous studies tested the amount of time spent using social media (e.g., [28]) or specific patterns, like problematic SMU (e.g., [42]), we suggest distinguishing between different patterns of uses, following the *digital Goldilocks hypothesis* [5] to investigate what pater may promote body image. In addition, while other studies link body image with a particular act of posting selfies on social media (e.g., [43]), we aim to examine whether communicating through social media can be associated with the risk of poor body image.

Therefore, our study aimed to investigate the associations between four types of SMU and adolescent body image using data from an extensive, cross-national survey across 42 European and North American countries and regions. We hypothesised that intensive SMU and problematic SMU would be associated with negative body image (negative subjective body weight) and over/underestimated body weight congruence, compared to non-active and active SMU. In addition, we expect these associations to be stronger for girls.

## Methods

### Sample and Procedure

The present study utilised data from the 2018 Health Behaviour in School-Aged Children (HBSC) survey. The HBSC is a World Health Organization (WHO) collaborative cross-national survey of adolescents’ health and health-related behaviours conducted every 4 years. Data were collected from each country following the HBSC internationally approved protocol to ensure consistency in instruments, data collection, and processing procedures [44].

**FIGURE 1:**
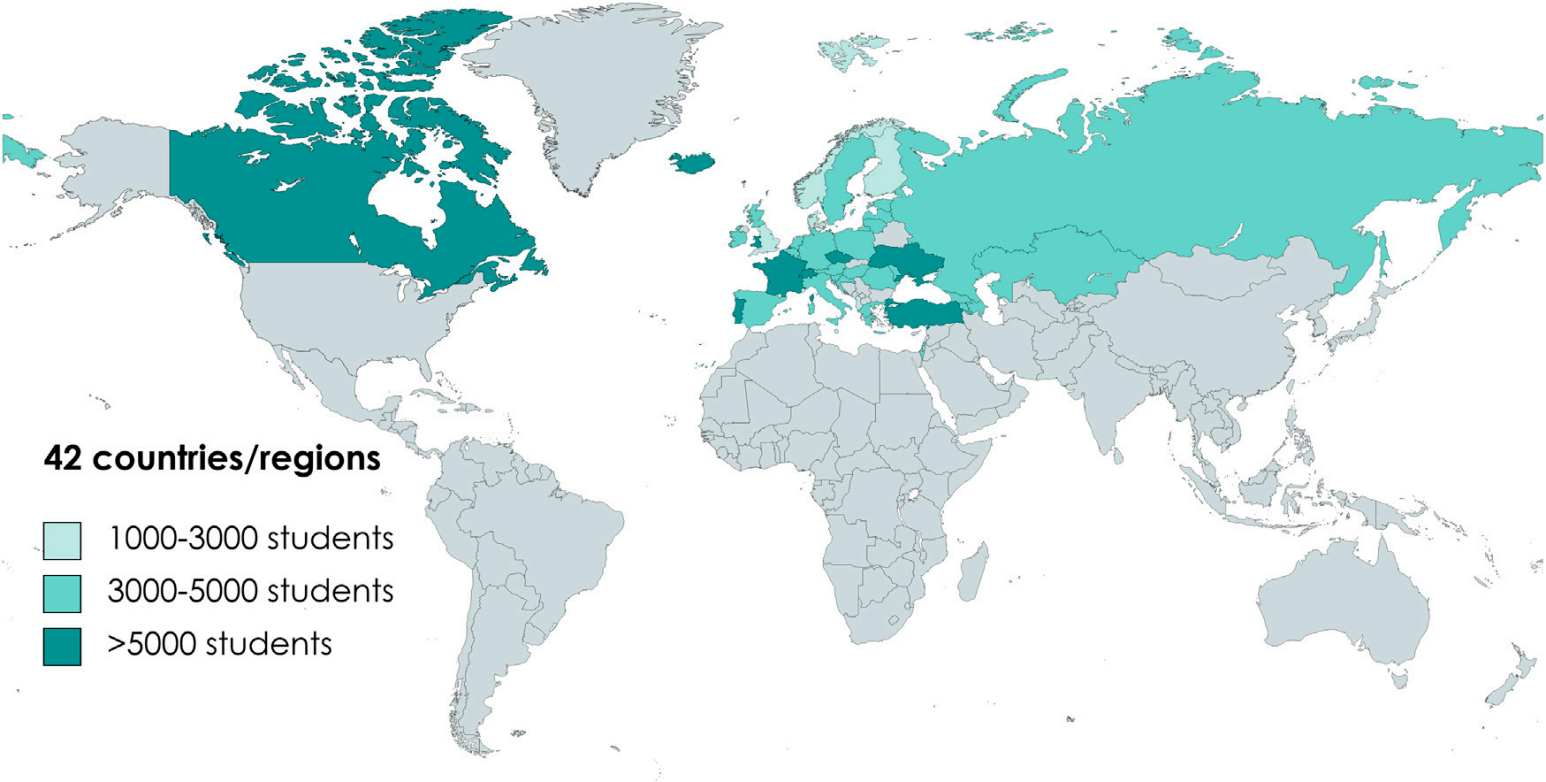
Sample size of countries and regions involved in the present study [Albania, Azerbaijan, Austria, Armenia, Belgium (Flemish), Belgium (French), Canada, Croatia, Czech Republic, Denmark, Estonia, Finland, France, Georgia, Germany, Greece, Hungary, Iceland, Ireland, Israel, Italy, Kazakhstan, Latvia, Lithuania, Luxembourg, Malta, Republic of Moldova, Netherlands, Norway, Poland, Portugal, Romania, Russia, Slovenia, Spain, Sweden, Switzerland, Turkey, Ukraine, England, Scotland, Wales. 2018].

Nationally representative samples of 11-, 13-, and 15-year-old pupils were recruited from classes within schools using a stratified systematic cluster sampling. Data were collected in classroom settings through standardised, self-filled questionnaires on health indicators, social background, and health-related behaviours. The study adhered to the principles of the Declaration of Helsinki, ensuring anonymity and confidentiality of all participants, and participation was voluntary. Ethical clearance was obtained by lead institutions in all HBSC member countries. Out of 47 countries participating in the 2018 HBSC wave, the present study included 42 countries and regions (Figure 1). The eligibility criteria for inclusion were (Supplementary Figure S1): SMU data availability (Bulgaria, Greenland, and Slovakia were excluded), cross-country comparability, and congruence of the collected subjective body weight measure (Macedonia and Serbia were excluded; for further details see Supplementary Figure S1), less than 40% missing values on Subjective Body Weight measure. Exclusively for the SMU-BWC association, having less than 40% missing values on Body Mass Index (BMI) was added as an additional eligibility criterion (Wales, Scotland, Ireland, and England were excluded), resulting in 38 countries/regions in the final analyses.

The overall sample from the 42 countries and regions included 223,454 pupils. Due to missing values on SMU usage categories, 32,562 (14.6%) questionnaires were excluded. Of 190,892 respondents, 51.5% were girls, 62.2% belonged to middle SES, and the distribution by age group was similar (29.0% 11 years old, 34.9% 13 years old, 36.0% 15 years old).

### Measures

*SMU Intensity* [45]. Participants identified how often they were in contact via social media with the following four categories of people—close friends, friends from a larger friend group, friends they met through the Internet, and other people (such as siblings or classmates). Five frequency options ranged from “(almost) never to” almost all the time throughout the day and were complemented by “do not know/does not apply” response. Data analysis included participants if at least one response from the four categories of people provided information. The highest frequency reported across the four items was used to create three levels of SMU intensity: a) never or at most weekly, b) daily/several times a day, and c) almost all the time.

*Problematic SMU*. The Social Media Disorder Scale [8] was used to identify participants displaying signs of possible problematic SMU. The scale showed good validity and reliability across countries [46]. The scale asked participants if they experienced nine items related to addictive behaviours during the past year using binary responses (yes/no): 1) regularly found that you cannot think of anything else but the moment that you will be able to use social media again; 2) regularly felt dissatisfied because you wanted to spend more time on social media; 3) often felt bad when you could not use social media; 4) tried to spend less time on social media, but failed 5) regularly neglected other activities (e.g., hobbies, sport); because you wanted to use social media; 6) regularly had arguments with others because of your social media use; 7) regularly lied to your parents or friends about the amount of time you spend on social media; 8) often used social media to escape from negative feelings; 9) had serious conflict with your parents, brother(s) or sister(s) because of your social media use. Following a validation study, participants who reported six or more symptoms were labelled problematic users, whereas those with five or fewer symptoms were labelled non-problematic users [46].

Four categories of SMU [6]. Four types of social media users were created based on their SMU and intensity scales: 1) non-active user (online contact with others not at all or at the most weekly AND non-problematic user); 2) active user (online contact with others daily but not all time AND non-problematic user); 3) intensive user (online contact almost all the time throughout the day AND non-problematic user); and 4) problematic user (six or more symptoms regardless of the intensity of SMU).

*Subjective Body Weight.* The self-perception of body image (Ojala and Kenny, 2018) was assessed by asking: Do you think your body is? With five possible responses recorded in three categories, combining extreme answers: below normal weight (much too slim” and “a bit too slim”), normal weight (“about the right size”), and overweight (“a bit too fat”, or “much too fat”). The item showed a high level of agreement in the test-retest analysis [47, 48].

*Body Mass Index (BMI).* Participants were asked to report on their height and weight. This was used to calculate the body mass index (BMI) for each participant using the formula (weight in kg)/(height in cm^2^). Participants were further categorised into four body mass categories (thin, normal, overweight, and obese) based on the World Health Organization’s classification criteria [49]. Self-reported height and weight are valid and acceptable proxies of the actual measurement, especially when the latter is not available [50–52].

Body Weight Congruence (BWC)*.* The congruence of Subjective Body Weight and body mass category (BMI) was evaluated by combining these two variables into three categories of BWC: 1) Correct weight perception (Group 0): normal BMI and thinking they are about right weight OR BMI below normal and think they are below normal weight OR overweight and obese BMI and think they are too fat; 2) Overestimation (Group 1): normal BMI and consider themselves too fat OR BMI below normal and consider themselves just right or too fat; and 3) Underestimation (Group 2): BMI above normal and consider themselves too thin or just right OR normal BMI and consider themselves too thin [13, 53].

*Sociodemographic variables.* Participants were asked to report their gender, and their birth month and year were used to determine their age. Socio-economic status (SES) was assessed using the Family Affluence Scale (FAS) version 3. FAS is a six-item index developed within the HBSC, recognised as a valid indicator of family wealth (Torsheim et al., 2016). Items include a) the number of cars in the family; b) the number of bathrooms in the household; c) the number of computers in the household; d) ownership of a dishwasher; e) having one’s bedroom; and f) the number of holidays spent abroad in the last 12 months prior. The item responses were summed and transformed into a fractional rank score for each country separately [54]. The score, which ranged from 0 to 1, was then categorised as low SES (0–.2), medium SES (0.21–0.8), and high SES (0.81–1) to account for economic differences across the countries [3].

### Statistical Analyses

Prevalence estimates of SMU, Subjective Body Weight, and BWC categories were assessed separately by gender for each country (Supplementary Figures S2–S7) and then by age and gender (Supplementary Table S1). Descriptive analyses were performed using the corrected weighted Pearson Chi-square statistic (Table 1). Due to the differences in the prevalence between boys and girls, all regression analyses were stratified by gender.

**TABLE 1:** Distribution of sociodemographic and Subjective Body Weight/Body Weight Congruence (BWC) characteristics (row %) by Social Media Use (SMU) categories (Worldwide. 2018).

	Overall proportion (*n* = 190,892, col%)	Social media use (SMU) categories (row %)				*p*-value
		Non-active	Active user	Intensive user	Problematic user	
Overall proportion (*n* = 190,892, row%)		(*n* = 29,634, 15.5%)	(*n* = 88,090, 46.2%)	(*n* = 59,281, 31.1%)	(*n* = 13887, 7.3%)	
Gender						
Boys	48.5	18.2	46.9	28.5	6.4	<0.001
Girls	51.5	13.4	45.7	32.9	8.0	
Age						
11 yrs	29	24.9	44.5	25.2	5.4	<0.001
13 yrs	34.9	14.0	47.4	30.9	7.8	
15 yrs	36	9.1	46.9	35.7	8.3	
Socio-economic status (SES)		(*n* = 28,691, 15.4%)	(*n* = 85,966, 46.2%)	(*n* = 57,795, 31.1%)	(*n* = 13485, 7.3%)	
Low	18.3	19.4	43.7	29.1	7.8	<0.001
Medium	62.2	15.4	47.3	30.4	6.9	
High	19.4	12.2	46.3	24.0	7.5	
Subjective body weight		(*n* = 29,208, 15.5%)	(*n* = 87,003, 46.2%)	(*n* = 58,447, 31.0%)	(*n* = 13686, 7.3%)	
Too thin	16.9	16.5	46.3	29.7	7.5	<0.001
About right weight	56.8	16.4	47.1	30.4	6.0	
Too fat	26.4	13.5	44.9	32.1	9.5	
Body weight congruence (BWC)		(*n* = 23,404, 15.4%)	(*n* = 71,121, 46.8%)	(*n* = 47,044, 30.9%)	(*n* = 10510, 6.9%)	
Underestimation	21.4	13.1	45.8	32.0	9.0	<0.001
Congruence	62.3	15.6	47.7	30.4	6.3	
Overestimation	16.3	16.8	46.2	30.0	6.9	

Abbreviations. SMU, social media use; yrs, years; SES, socio-economic status; BWC, body weight congruence.

^a^ Descriptive analyses based on 42 countries participating in the present study.

A set of multinomial logistic regression models was carried out to study the effect of SMU categories within each country on Subjective Body Weight and BWC separately and adjusted by age categories and SES (Supplementary Tables S2–S5). Finally, three-level multinomial regression models introducing the fixed effect played by pupils at the lowest level (level 1) and the two random effects played by schools (level 2) and countries (level 3) were performed through Generalized Structural Equation Modelling (GSEM). The multivariable associations of four categories of SMU with Subjective Body Weight and BWC, considered two separate dependent variables, were stratified by gender and adjusted by age categories and SES. In all the regression analyses, the reference categories were: active users for SMU categories, normal weight for Subjective Body Weight, correct weight perception for BWC, 11 years old for age, and low SES.

All the analyses were performed considering survey design effects (including stratification, clustering, and weighting) using STATA version 17.1 (StataCorp, College Station, TX). Because there were multiple comparisons, a more conservative approach to type 1 error was set, and the significance level of 1% was used.

## Results

### Descriptive

As shown in Table 1, active social media users (46.2%) were the most prevalent SMU group in our sample, followed by intensive users (31.1%), non-active users (15.5%), and problematic users (7.3%). More than half of respondents perceived themselves as about the right size (56.8%), 16.9% as too thin and 26.4% as too fat. About 2 out of 3 participants presented body weight congruence (62.3%), while 21.4% underestimated and 16.3% overestimated their weight.

Girls and older adolescents were classified more frequently than boys and the younger as intensive or problematic users. The relative proportion of intensive and problematic social media users was higher among those who perceived themselves as too fat than those about the right size. Similarly, higher rates of intensive (32.0%) or problematic (9.0%) SMU participants were observed in the BWC underestimation group compared to the body congruent one (30.4% and 6.3%, respectively). More detailed descriptive information on SMU, Subjective Body Weight, and BWC by country, age category and gender groups can be found in the Supplementary Figures S2–S7 and Supplementary Table S1.

### The Association Between SMU and Subjective Body Weight/BWC

**FIGURE 2:**
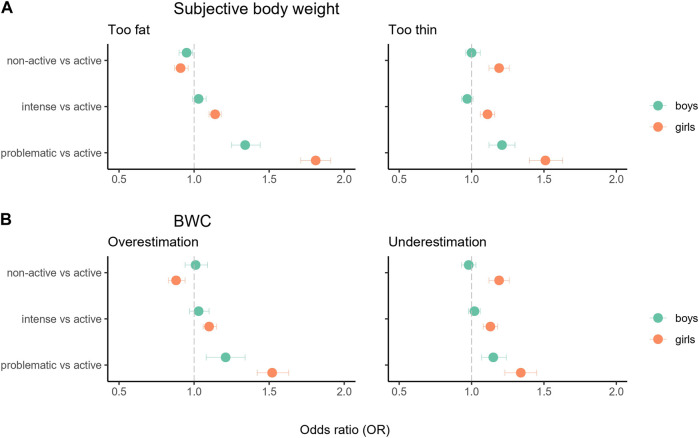
Association between SMU and Subjective Body Weight **(A)** and BWC **(B)** among girls and boys (Worldwide, 2018).

The association between SMU and Subjective Body Weight and BWC are shown in Tables 2, 3 and summarised in Figure 2. Overall, older age, lower socio-economic status and both intensive and problematic social media user profiles seem to play a risk role in the two explored body image outcomes (i.e., Subjective Body Weight and BWC), especially among girls. Concerning subjective body weight, intensive and problematic user girls were up to 80% more likely to perceive themselves as too fat (respectively, OR: 1.14; 95% CI: 1.10–1.18; OR: 1.81; 95% CI: 1.71–1.91) than active users, whereas among boys the positive relationship was significant only for problematic users (OR: 1.34; 95% CI: 1.25–1.44). A weaker positive association was found looking at the self-perception as too thin among intensive (OR: 1.11; 95% CI: 1.06–1.16) and problematic user girls (OR: 1.51; 95% CI: 1.40–1.63) and among problematic user boys (OR: 1.21; 95% CI: 1.12–1.30) compared to active users.

**TABLE 2:** Association between SMU and Subjective Body Weight (base outcome: perceiving the right weight) among girls and boys (Worldwide. 2018).

	Too fat				Too thin			
	Boys		Girls		Boys		Girls	
	OR	*p*	OR	*p*	OR	*p*	OR	*P*
SMU								
Active user (ref.)	1		1		1		1	
Non-active user	0.95	0.03	**0.91**	<0.001	1.00	0.85	**1.19**	<0.001
Intensive user	1.03	0.09	**1.14**	<0.001	0.97	0.19	**1.11**	<0.001
Problematic user	**1.34**	<0.001	**1.81**	<0.001	**1.21**	<0.001	**1.51**	<0.001
Age								
11 yrs (ref.)	1		1		1		1	
13 yrs	**1.18**	<0.001	**1.53**	<0.001	**1.07**	0.003	0.97	0.27
15 yrs	**1.13**	<0.001	**1.71**	<0.001	**1.32**	<0.001	0.94	0.02
Socio-economic status								
Low (ref.)	1		1		1		1	
Medium	**0.86**	<0.001	**0.81**	<0.001	**0.93**	0.001	**0.84**	<0.001
High	**0.71**	<0.001	**0.73**	<0.001	**0.89**	<0.001	**0.84**	<0.001

Model*	Boys				Girls			
	Coefficient		Standard error		Coefficient		Standard error	
Level 1 School Variance	0.07		0.009		0.08		0.110	
Level 2 Country Variance	0.06		0.014		0.05		0.110	

Note. In bold if *p* < 0.01.

^*^Three-level multinomial regression models introducing the fixed effect played by pupils at the lowest level (level 1) and the two random effects played by schools (level 2) and countries (level 3) were performed among girls and boys. The estimated variances of the two random effects are very small (<0.10), reaching statistical significance exclusively among boys.

Abbreviations. SMU, social media use; OR, odds ratio; *p*, *p*-value; Coeff, coefficient; S.E., standard error.

**TABLE 3:** Association between Social Media Use (SMU) and Body Weight Congruence (BWC) among girls and boys (Worldwide. 2018).

	Overestimation				Underestimation			
	Boys		Girls		Boys		Girls	
	OR	*p*	OR	*p*	OR	*p*	OR	*p*
SMU								
Active user (ref.)	1		1		1		1	
Non-active user	1.01	0.72	**0.88**	<0.001	0.98	0.47	**1.19**	<0.001
Intensive user	1.03	0.30	**1.10**	<0.001	1.02	0.33	**1.13**	<0.001
Problematic user	**1.21**	0.001	**1.52**	<0.001	**1.15**	**<0**.001	**1.34**	<0.001
Age								
11yrs o (ref.)	1		1		1		1	
13 yrs	**1.13**	<0.001	**1.27**	<0.001	**0.92**	<0.001	**0.79**	<0.001
15 yrs	0.95	0.14	**1.39**	<0.001	1.02	0.30	**0.72**	<0.001
Socio-economic status								
Low (ref.)	1		1		1		1	
Medium	1.03	0.41	1.00	0.93	**0.93**	0.001	**0.90**	0.001
High	1.01	0.79	1.03	0.30	**0.89**	<0.001	**0.85**	<0.001

Model*	Boys				Girls			
	Coeff		S.E.		Coeff		S.E.	
Level 1 School	0.04		0.01		0.19		0.02	
Level 2 country	0.02		0.01		0.23		0.02	

Note: In bold if *p* < 0.01.

^*^Three-level multinomial regression models introducing the fixed effect played by pupils at the lowest level (level 1) and the two random effects played by schools (level 2) and countries (level 3) were performed among girls and boys. The estimated variances of the two random effects are very small among boys (<0.10), while they are relevant among girls (range: 0.15–0.30), reaching statistical significance exclusively among girls.

Abbreviations. SMU, social media use; BWC, body weight congruence; OR, odds ratio; *p*, *p*-value; Coeff, coefficient; S.E., standard error.

The same significant positive pattern was also observed regarding BWC (Table 3), where both girls and boys with problematic SMU showed a higher likelihood, compared to active users, in terms of overestimation (+52% and +21% in mean, respectively) or underestimation (+34% and +15% in mean, respectively). Furthermore, intensive SMU girls were significantly more likely to present BWC overestimation (+10% in mean) and underestimation (+13% in mean).

The relationship between the non-active social media user profile (compared to the active user) and the two adopted body image measures (Subjective body weight and BWC) deserved a separate presentation of results. Regarding Subjective Body Weight, non-active users, among boys and girls, were about 5%–10% less likely to consider themselves as too fat (respectively, OR: 0.95, 95% CI: 0.90–0.99; OR: 0.91, 95% CI: 0.87–0.96) than active users, while non-active girls (but not boys) were about 20% more likely to think they were too thin (OR: 1.19, 95% CI: 1.12–1.26). Similarly to what was evidenced for Subjective Body Weight, exclusively among girls, non-active SMU was associated with a −12% mean likelihood of weight overestimation (OR: 0.88, 95% CI: 0.83–0.94) and a +19% mean likelihood of underestimation (OR: 1.19, 95% CI: 1.12–1.26).

## Discussion

The present study aimed to investigate the associations between four types of SMU and body image using data from an extensive, cross-national survey across 42 European and North American countries and regions. We hypothesised that intensive SMU and problematic SMU would be associated with negative body image (negative subjective body weight) and over/underestimated body weight congruence, compared to non-active and active SMU. In addition, we expect these associations to be stronger for girls.

Our study further supports the Sociocultural Theory, showing how different patterns of SMU act as a possible social agent for the development of a negative body. Overall, we found that intensive SMU and problematic SMU are associated with negative body image compared to active users. Non-active users were less likely to consider themselves too fat than active users. However, interesting findings point out that girls who are non-active social media users were also associated with a lower weight overestimation and a higher weight underestimation. The *Sociocultural Theory* elaborates on other agents, such as peers and parents and their role regarding the pressure to conform to unrealistic body ideals [16]. Therefore, the issue of non-active social media users requires further study.

In addition, the distinction between different patterns of SMU, following the *digital Goldilocks hypothesis* [5], got additional support in the current study, as findings showed how each pattern was associated with body image. Our findings reflected that boys and girls are at risk for negative body image, but not necessarily from the same SMU patterns. This distinction may help identify the risk for negative body image while testing the SMU. For instance, intensive and problematic social media users were more likely to perceive themselves as too fat or too thin than active social media users. However, the association was statistically significant among intensive and problematic girl users, whereas among boys, the relationship was significant only for problematic users. Both girls and boys with problematic SMU showed higher odds of overestimation or underestimation than active users. However, a statistically significant association was found among girls between intensive SMU and BWC overestimation and underestimation. Therefore, it seems that for boys, the problematic component of SMU is critical. The current study shows that the risk of negative body image occurs in boys with the problematic-addictive pattern of SMU.

Following previous cross-sectional studies (e.g., 28), our results support the hypothesis that problematic SMU may impede positive body image in adolescents. However, according to our research study, for girls also, intensive SMU is associated with negative body image. According to the *Differential Susceptibility to Media Effects Model* [55], media effects on an individual’s health and wellbeing depend on one’s susceptibility to media effects, including their dispositional (e.g., gender), developmental (e.g., age), and social (e.g., peer) susceptibility.

We found, among girls, a statistically significant association between intensive SMU and both BWC overestimation and underestimation. Following previous findings, girls were more likely to be problematic and intensive social media users [46]. In addition, girls were also more prone to poorer body image [56]. Previous studies found that exposure to idealised images on Instagram resulted in increased body dissatisfaction among young women regardless of the target girl (e.g., a peer known to participants, a same-aged girl peer that was unknown to participants, a famous girl Instagram influencer or a girl celebrity) [43, 57]. It is also known that the pressure to meet appearance ideals represented by thin and fit bodies is highly salient in young women and adolescent girls with frequent SMU [58, 59]. Consequently, young women tend to have a distorted perception of their body size and see their bodies as distant from appearance ideals [58].

### Study Strengths and Limitations

The present study has important strengths related to the large representative cross-national sample adopting a standard self-administered questionnaire. In addition, the study included a conceptual distinction between four patterns of SMU and two perceptions of body image.

Yet, the study has several limitations. First, the data were obtained using self-report questionnaires. Adolescents do not precisely estimate weight and height, resulting in BMI values that represent merely proxies of the actual measurement. Therefore, further studies are encouraged to use other measurement approaches (e.g., objective data and mixed method approaches) to obtain a more comprehensive picture of the positive and negative impacts of SMU on adolescent body image. Second, the study’s cross-sectional design does not allow for causal inferences. Thus, the present study cannot determine with certainty whether the outcomes observed are an effect of different patterns of SMU. Therefore, further longitudinal studies would be needed to test the direction of the associations. Third, some weight-related measures have a high percentage of missing values (up to 40%). Whether or not substantial differences were found among respondents and non-respondents by socio-economic status, our finding showed slight gender and age differences regarding SMU, subjective body weight and BWC measures. Fourth, a binary indicator of gender (boy vs. girl) did not reflect the experiences of adolescents whose identities do not match this binary conceptualisation. Future research on other underrepresented groups (e.g., minority identities related to gender and sexual orientation) is needed, as they may be characterised by unique experiences of SMU related to body image.

To conclude, the findings in the present study provide a more nuanced understanding of associations between SMU and body image within a large, cross-national adolescent population and highlight the potential risks of intensive and problematic SMU to adolescent self-perceptions. In particular, high exposure to representations of body ideals on social media and increased vulnerability to social feedback and social comparison may harm the development of a healthy body image during adolescence. The findings from this study highlight the need for policymakers, educators, parents, clinicians, and others who care for adolescents to be aware of the associations between SMU patterns and adolescent body image.

On a more practical level, the findings suggest that interventions and preventive programs should be tailored to adolescents’ specific patterns of social media use. More specifically, intensive and problematic use of social media may play the most crucial role in explaining its detrimental consequences for negative body image and over/underestimated body weight congruence. Thus, interventions should enable a shift from maladaptive to harmonious engagement, for example, by supporting adolescents to evaluate contents related to body appearances or beauty standards critically (e.g., idealise thinness, fitness, and esthetical perfection) and adjust their own media consumption (e.g., media literacy) [60] establish and maintain functionality appreciation (a component of body neutrality) by focusing on appreciate the body for the functions it performs (e.g., physical capacities, senses and sensations, creativity, communication with others, and self-care) despite appearance satisfaction [61].
