# Supplementary material for: Different Categories of Social Media Use and Their Association With Body Image Among Adolescents in 42 Countries
Source: Int J Public Health. 2024 Jul 3;69:1606944. doi: 10.3389/ijph.2024.1606944 (PMC11252497; doi:10.3389/ijph.2024.1606944)
Supplement: Supplementary file 1 [file DataSheet1.docx]

**Different Patterns of Social Media Use and Their Association with Body Image Among Adolescents in 42 Countries**

**Supplementary**

**Figure S1 Flow diagram of the selection of countries and regions in the present work.**

**
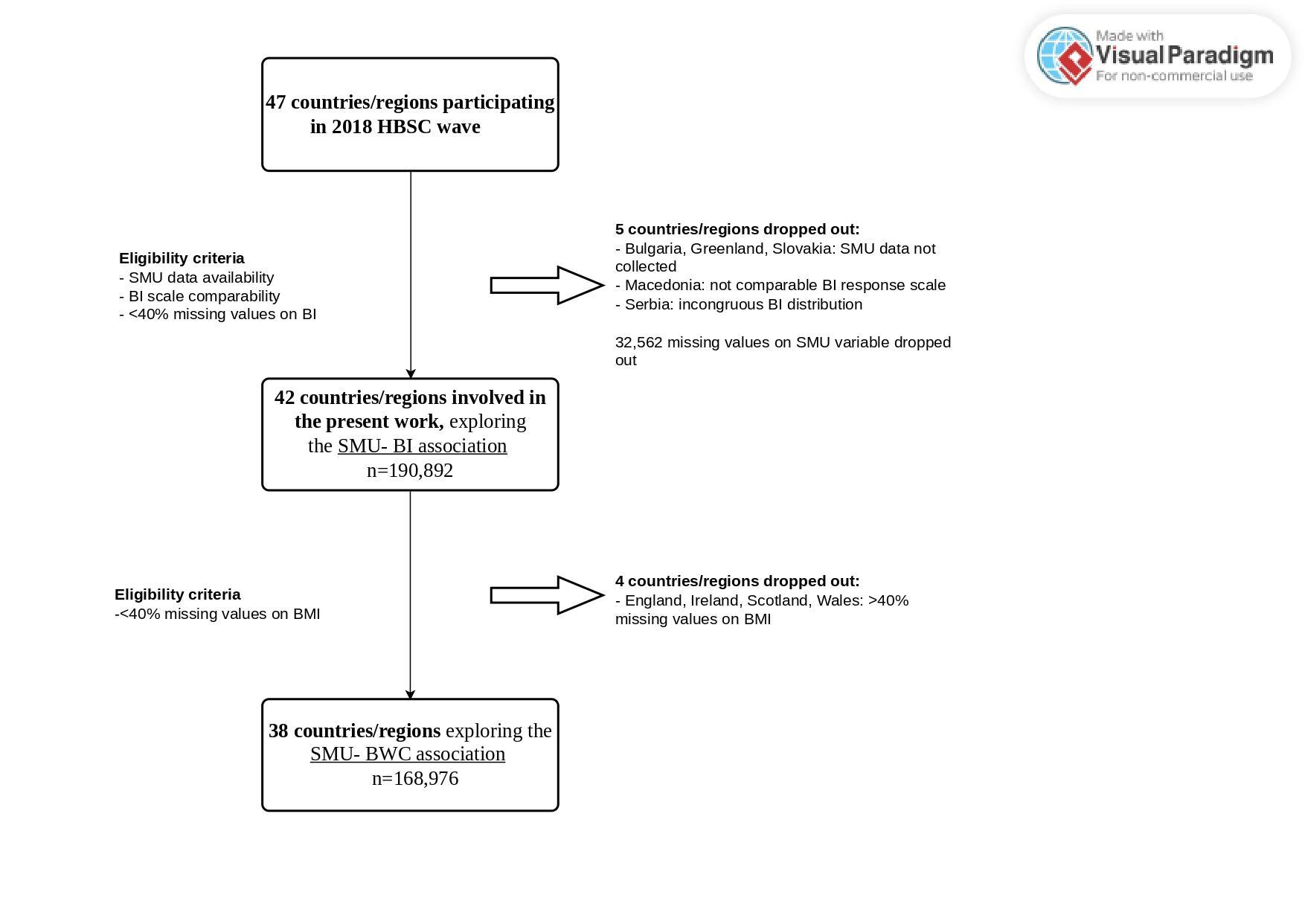
**

**Figure S2 Social media use (SMU) distribution in the 42 involved HBSC countries among boys (2018).**

Abbreviations. A.L., Albania; AM, Armenia; AT, Austria; AZ, Azerbaijan; BE-VLG, Belgium (French); BE-WAL, Belgium (Flemish), CA, Canada; C.H., Switzerland; CZ, Czech Republic; DE, Germany; DK, Denmark; E.E. Estonia; ENG, England; ES, Spain; FI, Finland; FR, France; GE, Georgia, GR, Greece; H.R., Croatia; H.U., Hungary; IE, Ireland; IL, Israel; I.S., Iceland; I.T., Italy; K.Z., Kazakhstan; L.T., Lithuania; LU, Luxemburg; LV, Latvia; M.D. Republic of Moldova; MT, Malta; N.L., Netherlands; NO, Norway; PL, Poland; PT, Portugal; R.O., Romania; R.U., Russia; SCT, Scotland; SE, Sweden; S.I., Slovenia; T.R., Turkey; U.A., Ukraine; WLS, Wales.

**Figure S3 Social media use (SMU) distribution in the 42 involved HBSC countries among girls (2018).**

Abbreviations. A.L., Albania; AM, Armenia; AT, Austria; AZ, Azerbaijan; BE-VLG, Belgium (French); BE-WAL, Belgium (Flemish), CA, Canada; C.H., Switzerland; CZ, Czech Republic; DE, Germany; DK, Denmark; E.E. Estonia; ENG, England; ES, Spain; FI, Finland; FR, France; GE, Georgia, GR, Greece; H.R., Croatia; H.U., Hungary; IE, Ireland; IL, Israel; I.S., Iceland; I.T., Italy; K.Z., Kazakhstan; L.T., Lithuania; LU, Luxemburg; LV, Latvia; M.D. Republic of Moldova; MT, Malta; N.L., Netherlands; NO, Norway; PL, Poland; PT, Portugal; R.O., Romania; R.U., Russia; SCT, Scotland; SE, Sweden; S.I., Slovenia; T.R., Turkey; U.A., Ukraine; WLS, Wales.

**Figure S4 Subjective Body Weight distribution in the 42 involved HBSC countries among boys (2018).**

Abbreviations. A.L., Albania; AM, Armenia; AT, Austria; AZ, Azerbaijan; BE-VLG, Belgium (French); BE-WAL, Belgium (Flemish), CA, Canada; C.H., Switzerland; CZ, Czech Republic; DE, Germany; DK, Denmark; E.E. Estonia; ENG, England; ES, Spain; FI, Finland; FR, France; GE, Georgia, GR, Greece; H.R., Croatia; H.U., Hungary; IE, Ireland; IL, Israel; I.S., Iceland; I.T., Italy; K.Z., Kazakhstan; L.T., Lithuania; LU, Luxemburg; LV, Latvia; M.D. Republic of Moldova; MT, Malta; N.L., Netherlands; NO, Norway; PL, Poland; PT, Portugal; R.O., Romania; R.U., Russia; SCT, Scotland; SE, Sweden; S.I., Slovenia; T.R., Turkey; U.A., Ukraine; WLS, Wales.

**Figure S5 Subjective Body Weight distribution in the 42 involved HBSC countries among girls (2018).**

Abbreviations. A.L., Albania; AM, Armenia; AT, Austria; AZ, Azerbaijan; BE-VLG, Belgium (French); BE-WAL, Belgium (Flemish), CA, Canada; C.H., Switzerland; CZ, Czech Republic; DE, Germany; DK, Denmark; E.E. Estonia; ENG, England; ES, Spain; FI, Finland; FR, France; GE, Georgia, GR, Greece; H.R., Croatia; H.U., Hungary; IE, Ireland; IL, Israel; I.S., Iceland; I.T., Italy; K.Z., Kazakhstan; L.T., Lithuania; LU, Luxemburg; LV, Latvia; M.D. Republic of Moldova; MT, Malta; N.L., Netherlands; NO, Norway; PL, Poland; PT, Portugal; R.O., Romania; R.U., Russia; SCT, Scotland; SE, Sweden; S.I., Slovenia; T.R., Turkey; U.A., Ukraine; WLS, Wales.

**Figure S6 Body weight congruence (BWC) distribution in the 42 involved HBSC countries among boys (2018).**

Abbreviations. A.L., Albania; AM, Armenia; AT, Austria; AZ, Azerbaijan; BE-VLG, Belgium (French); BE-WAL, Belgium (Flemish), CA, Canada; C.H., Switzerland; CZ, Czech Republic; DE, Germany; DK, Denmark; E.E. Estonia; ENG, England; ES, Spain; FI, Finland; FR, France; GE, Georgia, GR, Greece; H.R., Croatia; H.U., Hungary; IE, Ireland; IL, Israel; I.S., Iceland; I.T., Italy; K.Z., Kazakhstan; L.T., Lithuania; LU, Luxemburg; LV, Latvia; M.D. Republic of Moldova; MT, Malta; N.L., Netherlands; NO, Norway; PL, Poland; PT, Portugal; R.O., Romania; R.U., Russia; SCT, Scotland; SE, Sweden; S.I., Slovenia; T.R., Turkey; U.A., Ukraine; WLS, Wales.

**Figure S7 Body weight congruence (BWC) distribution in the 42 involved HBSC countries among girls (2018).**

Abbreviations. A.L., Albania; AM, Armenia; AT, Austria; AZ, Azerbaijan; BE-VLG, Belgium (French); BE-WAL, Belgium (Flemish), CA, Canada; C.H., Switzerland; CZ, Czech Republic; DE, Germany; DK, Denmark; E.E. Estonia; ENG, England; ES, Spain; FI, Finland; FR, France; GE, Georgia, GR, Greece; H.R., Croatia; H.U., Hungary; IE, Ireland; IL, Israel; I.S., Iceland; I.T., Italy; K.Z., Kazakhstan; L.T., Lithuania; LU, Luxemburg; LV, Latvia; M.D. Republic of Moldova; MT, Malta; N.L., Netherlands; NO, Norway; PL, Poland; PT, Portugal; R.O., Romania; R.U., Russia; SCT, Scotland; SE, Sweden; S.I., Slovenia; T.R., Turkey; U.A., Ukraine; WLS, Wales.

| \| **Table S1 Subjective Body Weight and body weight congruence (BWC) stratified by gender and age groups (42 countries. 2018).** \| \| \| \| \| \| \| \| \| --- \| --- \| --- \| --- \| --- \| --- \| --- \| --- \| \|  \| **Gender** \| \| \| **Age** \| \| \| \| \|  \| **Boys** \| **Girls** \| **p*** \| **11 yrs** \| **13 yrs** \| **15 yrs** \| **p*** \| \| **Subjective body weight** \|  \|  \|  \|  \|  \|  \|  \| \| Too thin \| 19.5 \| 13.3 \| <0.001 \| 16.4 \| 15.6 \| 16.3 \| <0.001 \| \| About right weight \| 57.8 \| 55.1 \| 61.0 \| 55.5 \| 53.2 \| \| Too fat \| 22.7 \| 31.6 \| 22.6 \| 28.9 \| 27.4 \| \| **Body weight congruence (BWC)** \|  \|  \|  \|  \|  \|  \|  \| \| Underestimation \| 27.1 \| 16.0 \| <0.001 \| 23.3 \| 20.5 \| 20.8 \| <0.001 \| \| Congruence \| 62.5 \| 62.2 \| 62.9 \| 62.5 \| 61.7 \| \| Overestimation \| 10.4 \| 21.8 \| 13.8 \| 17.0 \| 17.5 \| \| *Pearson test corrected for study design was performed. \| \| \| \| \| \| \| \| \| Abbreviations. BWC, body weight congruence. \| \| \| \| \| \| \| \| \|  \|  \|  \|  \|  \|  \|  \|  \|     **Table S2 Association between SMU (reference: active users) and subjective Body Weight (base outcome: perceiving about right weight) by country among boys (42 countries. 2018).** | | | | | | |
| --- | --- | --- | --- | --- | --- | --- | --- | --- | --- | --- | --- | --- | --- | --- | --- | --- | --- | --- | --- | --- | --- | --- | --- | --- | --- | --- | --- | --- | --- | --- | --- | --- | --- | --- | --- | --- | --- | --- | --- | --- | --- | --- | --- | --- | --- | --- | --- | --- | --- | --- | --- | --- | --- | --- | --- | --- | --- | --- | --- | --- | --- | --- | --- | --- | --- | --- | --- | --- | --- | --- | --- | --- | --- | --- | --- | --- | --- | --- | --- | --- | --- | --- | --- | --- | --- | --- | --- | --- | --- | --- | --- | --- | --- | --- | --- | --- | --- | --- | --- | --- | --- | --- | --- | --- | --- | --- | --- | --- | --- | --- |
|  | **Being fat** | | | **Being thin** | | |
|  | **SMU (ref. active user)** | | | **SMU (ref. active user)** | | |
| **Country** | **non-active** | **intense** | **problematic** | **non-active** | **intense** | **problematic** |
| **AL** | 1.06 | 1.55 | 0.39 | 1.14 | 1.27 | 0.73 |
| **AM** | 1.17 | 1.26 | 1.37 | 1.14 | 1.15 | 1.45 |
| **AT** | 0.85 | 1.03 | 1.19 | 0.93 | **1.32** | 1.42 |
| **AZ** | 0.87 | 1.35 | **0.21*** | 0.86 | 0.88 | **0.26*** |
| **BE-VLG** | 0.98 | 1.17 | **2.63*** | 1.12 | 0.92 | 1.05 |
| **BE-WAL** | 0.92 | 0.90 | 1.00 | 0.96 | 0.96 | **1.57** |
| **CA** | 1.01 | 1.30 | 2.12 | 0.77 | 1.15 | **2.40*** |
| **CH** | 0.82 | 1.23 | 1.55 | 0.84 | 0.84 | **1.59** |
| **CZ** | 0.91 | 0.88 | 1.25 | 1.09 | 0.99 | 1.09 |
| **DE** | 0.78 | 1.00 | 1.50 | 0.74 | 1.11 | 0.92 |
| **DK** | 1.40 | 0.99 | 1.81 | 0.84 | 0.97 | **2.35** |
| **EE** | 0.95 | **0.76** | **1.85*** | 0.98 | **0.78** | **1.68** |
| **ENG** | 1.12 | 1.55 | 0.91 | 1.10 | 1.27 | 1.35 |
| **ES** | 1.06 | 1.17 | 1.17 | 0.78 | 0.94 | 0.91 |
| **FI** | 1.39 | 1.26 | **2.24*** | 0.99 | 1.28 | 1.68 |
| **FR** | 0.96 | **0.70** | **2.99*** | 1.04 | 0.81 | 1.68 |
| **GE** | 1.36 | 1.29 | **1.96** | 0.98 | 0.91 | 0.98 |
| **GR** | 1.05 | 1.12 | 1.48 | 1.04 | 1.14 | **1.60** |
| **HR** | 0.98 | 0.92 | 0.87 | 1.09 | 0.86 | 0.70 |
| **HU** | 0.88 | 1.06 | 0.97 | 0.67 | 0.85 | 1.12 |
| **IE** | 1.14 | 1.00 | **1.54** | 0.97 | 0.98 | 1.55 |
| **IL** | 1.23 | 1.18 | **1.95** | 1.26 | 0.95 | 1.38 |
| **IS** | 0.93 | 1.07 | **2.30*** | 0.74 | 1.01 | 1.12 |
| **IT** | 0.99 | 1.28 | **2.14*** | 0.81 | 0.87 | 1.51 |
| **KZ** | 1.22 | 1.08 | 1.84 | 0.85 | 0.95 | 0.83 |
| **LT** | 1.07 | 0.90 | 0.82 | 0.99 | 0.92 | 1.06 |
| **LU** | 0.88 | 1.13 | 1.28 | 1.17 | 0.98 | 0.70 |
| **LV** | 1.11 | 1.10 | **1.90** | 0.81 | 0.85 | 0.67 |
| **MD** | 1.03 | 0.93 | 1.59 | 1.09 | 1.00 | 0.88 |
| **MT** | 0.75 | 0.70 | 1.43 | 1.08 | 1.26 | **2.00*** |
| **NL** | 1.19 | 1.17 | 0.87 | 1.12 | 0.86 | 1.81 |
| **NO** | 1.24 | 1.20 | 1.56 | 0.94 | 0.90 | 1.07 |
| **PL** | 0.85 | 0.90 | **1.80*** | 0.86 | 1.03 | **1.65** |
| **PT** | 0.92 | 1.06 | **1.55** | 0.86 | 0.86 | 0.67 |
| **RO** | 1.24 | 1.10 | 0.98 | 1.10 | 0.95 | 1.11 |
| **RU** | 0.77 | 0.99 | 1.09 | 1.09 | 0.90 | 1.45 |
| **SCT** | 0.74 | 1.03 | 0.76 | 1.12 | 1.02 | 0.80 |
| **SE** | 1.07 | 0.96 | 1.66 | 0.96 | 0.90 | **2.51*** |
| **SI** | 1.04 | 0.93 | 0.95 | 0.97 | 0.86 | 1.08 |
| **TR** | 1.01 | 1.10 | 1.34 | 0.97 | 1.04 | 0.95 |
|  |  |  |  |  |  |  |
| **UA** | 1.20 | **1.52*** | 1.52 | 1.01 | 1.04 | 0.90 |
| **WLS** | 0,95 | 0,89 | 1,12 | 0,82 | 0,86 | 1,24 |
| In bold if p<0.05; * if p<0.01 | | | | | | |
| Abbreviations. A.L., Albania; AM, Armenia; AT, Austria; AZ, Azerbaijan; BE-VLG, Belgium (French); BE-WAL, Belgium (Flemish), CA, Canada; C.H., Switzerland; CZ, Czech Republic; DE, Germany; DK, Denmark; E.E. Estonia; ENG, England; ES, Spain; FI, Finland; FR, France; GE, Georgia, GR, Greece; H.R., Croatia; H.U., Hungary; IE, Ireland; IL, Israel; I.S., Iceland; I.T., Italy; K.Z., Kazakhstan; L.T., Lithuania; LU, Luxemburg; LV, Latvia; M.D. Republic of Moldova; MT, Malta; N.L., Netherlands; NO, Norway; PL, Poland; PT, Portugal; R.O., Romania; R.U., Russia; SCT, Scotland; SE, Sweden; S.I., Slovenia; T.R., Turkey; U.A., Ukraine; WLS, Wales. | | | | | | |

| **Table S3 Association between SMU (reference: active users) and subjective Body Weight (base outcome: perceiving about right weight) by country among girls (42 countries. 2018).** | | | | | | |
| --- | --- | --- | --- | --- | --- | --- |
|  | **Being fat** | | | **Being thin** | | |
|  | **SMU (ref. active user)** | | | **SMU (ref. active user)** | | |
| **Country** | **non-active** | **intense** | **problematic** | **non-active** | **intense** | **problematic** |
| **AL** | 1.30 | 0.88 | 1.29 | 1.63 | 0.85 | 1.82 |
| **AM** | 1.02 | 1.03 | 1.91 | 1.02 | 1.15 | 1.65 |
| **AT** | 0.87 | **1.35*** | **2.37*** | 0.97 | 0.97 | 1.09 |
| **AZ** | 1.23 | 1.28 | 0.78 | 1.13 | 0.82 | **0.48*** |
| **BE-VLG** | 0.97 | 1.08 | **2.69*** | 1.18 | 0.85 | **2.33*** |
| **BE-WAL** | 0.74 | 0.85 | 1.09 | 1.14 | 1.10 | 1.50 |
| **CA** | 1.17 | 1.02 | **2.02*** | 0.75 | 0.83 | 1.64 |
| **CH** | 0.90 | **1.27** | **2.08*** | 0.89 | **1.70*** | **1.77** |
| **CZ** | 0.89 | **1.31*** | **2.07*** | 1.09 | **1.27** | **1.72*** |
| **DE** | 0.91 | **1.33*** | **2.24*** | 1.27 | 1.25 | **2.23** |
| **DK** | 0.96 | 1.13 | 1.47 | 0.91 | **1.56** | 1.97 |
| **EE** | 1.07 | 1.15 | 1.33 | 1.20 | 1.21 | 1.54 |
| **ENG** | 1.24 | 1.26 | **1.68** | 1.91 | 0.97 | 1.04 |
| **ES** | 1.03 | 0.99 | **1.63*** | 0.74 | 0.94 | **1.87*** |
| **FI** | 1.30 | **1.63*** | **2.08*** | 0.91 | 1.59 | 1.81 |
| **FR** | 1.03 | 1.23 | **1.76*** | **0.60** | 0.80 | 1.20 |
| **GE** | 0.98 | 1.00 | **2.22*** | 0.90 | 1.15 | 1.35 |
| **GR** | 1.00 | 1.22 | **1.94*** | 1.10 | **1.38** | 1.45 |
| **HR** | 1.22 | 1.13 | **1.75*** | 0.93 | 1.10 | **1.96*** |
| **HU** | **0.64** | 1.06 | **2.15*** | 1.34 | 1.29 | **2.23** |
| **IE** | 0.83 | 1.19 | **2.21*** | 0.56 | 0.97 | **2.36*** |
| **IL** | 1.45 | **1.32** | **2.84*** | 1.23 | 1.01 | 1.93 |
| **IS** | 0.93 | **1.49*** | **2.21*** | 0.62 | 1.00 | **2.22*** |
| **IT** | 1.57 | **1.28** | **1.86*** | 1.81 | **1.51** | 1.55 |
| **KZ** | **1.93*** | 1.28 | **4.40*** | 1.30 | **1.55*** | 1.65 |
| **LT** | 1.24 | 0.99 | 1.37 | 1.39 | 0.85 | 1.18 |
| **LU** | 1.02 | 1.03 | **2.21*** | 1.38 | **1.54*** | **1.91** |
| **LV** | 0.79 | **1.27** | **2.18*** | 1.01 | 1.36 | 1.86 |
| **MD** | 1.09 | 1.08 | **1.85*** | 0.83 | 0.95 | 1.02 |
| **MT** | 0.37 | 1.03 | 1.46 | 0.98 | 1.07 | 1.10 |
| **NL** | 0.77 | 1.16 | 1.61 | 1.39 | 1.11 | 1.73 |
| **NO** | 0.76 | **1.44** | 1.15 | 1.17 | 1.20 | 0.74 |
| **PL** | 0.99 | **0.80** | 0.39 | 0.94 | 1.12 | 0.70 |
| **PT** | 0.93 | 0.95 | **1.65*** | 0.95 | 0.98 | 1.05 |
| **RO** | 1.16 | **1.31** | **1.76*** | 1.38 | 1.22 | 1.47 |
| **RU** | **0.57*** | 1.03 | 1.26 | 0.94 | 1.08 | 1.56 |
| **SCT** | 1.18 | 1.17 | **1.68** | 0.58 | 0.97 | 1.92 |
| **SE** | 1.29 | 1.21 | **1.98*** | 0.73 | 1.16 | **2.19*** |
| **SI** | 0.91 | 1.06 | **2.03*** | 0.94 | 1.05 | 1.14 |
| **TR** | 1.10 | 1.08 | **1.59*** | 0.94 | 0.93 | 1.04 |
| **UA** | 0.92 | 1.14 | **1.89*** | **1.53** | 1.32 | **1.85*** |
| **WLS** | 1.03 | 0.94 | **1.55*** | 1.09 | 1.16 | 1.46 |
| In bold if p<0.05; * if p<0.01 | | | | | | |
| Abbreviations. A.L., Albania; AM, Armenia; AT, Austria; AZ, Azerbaijan; BE-VLG, Belgium (French); BE-WAL, Belgium (Flemish), CA, Canada; C.H., Switzerland; CZ, Czech Republic; DE, Germany; DK, Denmark; E.E. Estonia; ENG, England; ES, Spain; FI, Finland; FR, France; GE, Georgia, GR, Greece; H.R., Croatia; H.U., Hungary; IE, Ireland; IL, Israel; I.S., Iceland; I.T., Italy; K.Z., Kazakhstan; L.T., Lithuania; LU, Luxemburg; LV, Latvia; M.D. Republic of Moldova; MT, Malta; N.L., Netherlands; NO, Norway; PL, Poland; PT, Portugal; R.O., Romania; R.U., Russia; SCT, Scotland; SE, Sweden; S.I., Slovenia; T.R., Turkey; U.A., Ukraine; WLS, Wales. | | | | | | |

| **Table S4 Association between SMU (reference: active users) and BWC (base outcome: body weight congruence) by country among boys (42 countries. 2018).** | | | | | | |
| --- | --- | --- | --- | --- | --- | --- |
|  | **Overestimation** | | | **Underestimation** | | |
|  | **SMU (ref. active user)** | | | **SMU (ref. active user)** | | |
| **Country** | **non-active** | **intense** | **problematic** | **non-active** | **intense** | **problematic** |
| **AL** | 0.60 | 1.20 | 0.02 | 0.94 | 0.93 | 1.41 |
| **AM** | 1.45 | 1.21 | 1.66 | 1.26 | 1.33 | 1.45 |
| **AT** | 1.16 | 1.06 | 0.84 | 0.94 | **1.34** | 1.43 |
| **AZ** | 1.34 | 1.47 | 0.79 | **0.55*** | 0.78 | 0.76 |
| **BE-VLG** | 0.78 | 1.07 | 1.49 | 0.86 | 0.81 | 0.94 |
| **BE-WAL** | 0.85 | 0.91 | 1.08 | 0.94 | 1.26 | **1.93** |
| **CA** | 1.20 | 0.71 | 1.59 | 0.91 | 1.20 | 1.52 |
| **CH** | 1.00 | 1.11 | 1.21 | 0.87 | 1.04 | 1.19 |
| **CZ** | 1.16 | 0.91 | 1.33 | **1.22** | 1.18 | 1.41 |
| **DE** | 0.90 | 0.77 | 1.30 | 1.03 | 1.24 | 1.01 |
| **DK** | 1.43 | 1.06 | 1.39 | 1.31 | 0.96 | 1.81 |
| **EE** | 1.26 | 0.82 | 1.30 | 1.10 | 0.90 | 1.26 |
| **ES** | 0.98 | 0.87 | 0.80 | 0.81 | 0.78 | 0.73 |
| **FI** | 1.17 | 1.30 | **2.03** | **0.55** | 1.36 | 1.04 |
| **FR** | 1.14 | 0.93 | 1.26 | 0.81 | 0.97 | 1.21 |
| **GE** | 1.24 | **1.56** | **3.31*** | 1.00 | 0.91 | 1.33 |
| **GR** | 0.52 | 0.91 | 1.17 | 0.96 | 1.05 | 1.33 |
| **HR** | 0.76 | 0.85 | 0.76 | 1.16 | 0.96 | 0.82 |
| **HU** | 0.67 | 0.70 | 1.51 | 0.77 | 1.08 | 1.46 |
| **IL** | 1.10 | 1.09 | 2.04 | 1.24 | 1.09 | **1.97*** |
| **IS** | 0.77 | 0.75 | 1.26 | 1.01 | 1.17 | 0.76 |
| **IT** | 1.61 | 1.42 | 1.76 | 1.02 | 0.92 | 1.10 |
| **KZ** | 1.09 | 0.98 | 1.06 | 0.97 | 0.80 | 1.31 |
| **LT** | 1.23 | 1.09 | 1.31 | 0.90 | 0.94 | 0.85 |
| **LU** | 1.48 | 1.27 | 0.74 | 1.10 | 1.16 | 0.75 |
| **LV** | 0.94 | 1.07 | 1.71 | 0.78 | 0.91 | **0.47** |
| **MD** | 0.95 | 0.92 | 1.02 | 1.00 | 1.28 | 0.87 |
| **MT** | 0.70 | 1.32 | 0.46 | 1.36 | 2.00 | 1.01 |
| **NL** | 1.13 | 0.88 | 1.16 | 0.90 | 0.83 | 1.29 |
| **NO** | 1.36 | 0.82 | 1.79 | 1.08 | 0.91 | 1.66 |
| **PL** | 0.88 | 0.92 | **2.03*** | 1.01 | 1.02 | 1.43 |
| **PT** | 0.89 | 1.07 | 1.57 | 0.73 | 0.84 | 0.65 |
| **RO** | 1.00 | 1.00 | 0.63 | 0.96 | 0.94 | 1.07 |
| **RU** | 1.43 | 1.27 | **2.26*** | 1.27 | 0.85 | 1.50 |
| **SE** | 1.15 | 0.95 | **2.50** | 0.97 | 1.08 | **2.25*** |
| **SI** | 1.04 | 0.88 | 1.09 | 0.89 | 0.89 | 1.09 |
| **TR** | 1.53 | 1.20 | 0.82 | 1.10 | 0.98 | 1.04 |
| **UA** | 1.08 | 1.30 | **1.87*** | 1.14 | 0.92 | 0.96 |
| In bold if p<0.05; * if p<0.01 | | | | | | |
| Abbreviations. A.L., Albania; AM, Armenia; AT, Austria; AZ, Azerbaijan; BE-VLG, Belgium (French); BE-WAL, Belgium (Flemish), CA, Canada; C.H., Switzerland; CZ, Czech Republic; DE, Germany; DK, Denmark; E.E. Estonia; ES, Spain; FI, Finland; FR, France; GE, Georgia, GR, Greece; H.R., Croatia; H.U., Hungary; IL, Israel; I.S., Iceland; I.T., Italy; K.Z., Kazakhstan; L.T., Lithuania; LU, Luxemburg; LV, Latvia; M.D. Republic of Moldova; MT, Malta; N.L., Netherlands; NO, Norway; PL, Poland; PT, Portugal; R.O., Romania; R.U., Russia; SE, Sweden; S.I., Slovenia; T.R., Turkey; U.A., Ukraine. | | | | | | |

| **Table S5 Association between SMU (reference: active users) and BWC (base outcome: body weight congruence) by country among girls (42 countries. 2018).** | | | | | | |
| --- | --- | --- | --- | --- | --- | --- |
|  | **Overestimation** | | | **Underestimation** | | |
|  | **SMU (ref. active user)** | | | **SMU (ref. active user)** | | |
| **Country** | **non-active** | **intense** | **problematic** | **non-active** | **intense** | **problematic** |
| **AL** | 0.96 | 0.71 | 1.03 | 1.21 | 0.84 | **2.07** |
| **AM** | 1.11 | 1.07 | **2.60*** | 1.34 | 1.31 | 1.40 |
| **AT** | 1.28 | **1.34** | **1.89*** | 1.07 | 1.28 | 0.86 |
| **AZ** | 1.34 | 1.24 | 0.91 | 1.27 | 0.79 | **1.86** |
| **BE-VLG** | 0.98 | 1.03 | **2.18*** | 1.26 | 1.00 | 1.60 |
| **BE-WAL** | 0.76 | 1.00 | 0.96 | 1.03 | **1.56*** | 0.94 |
| **CA** | 1.31 | 0.99 | 1.57 | 0.89 | 0.77 | 0.55 |
| **CH** | 0.91 | 1.22 | 1.34 | 0.86 | **1.62*** | 1.35 |
| **CZ** | 0.91 | 1.21 | **1.89*** | 1.13 | 1.22 | **1.71*** |
| **DE** | 0.80 | 1.13 | **1.88*** | 1.22 | 1.19 | 1.17 |
| **DK** | 1.26 | 1.14 | 0.67 | 1.40 | 1.33 | 0.97 |
| **EE** | 0.82 | 0.91 | 0.96 | 1.17 | 1.07 | 1.25 |
| **ES** | 1.03 | 1.02 | 1.19 | 0.92 | 1.05 | 1.28 |
| **FI** | 1.52 | **1.55*** | 1.52 | 1.08 | 1.45 | 1.39 |
| **FR** | 0.88 | 1.02 | 1.16 | 0.64 | 0.90 | 0.76 |
| **GE** | 1.15 | 1.26 | 1.32 | 1.10 | 1.30 | 0.79 |
| **GR** | 0.76 | 1.17 | **1.56** | 0.95 | 1.12 | 1.05 |
| **HR** | 1.16 | 1.28 | **1.78*** | 0.97 | 1.15 | **1.78*** |
| **HU** | **0.38*** | 0.93 | **1.82*** | 1.10 | 1.16 | 1.21 |
| **IL** | 1.01 | 1.02 | **2.83*** | 1.06 | 0.80 | 1.14 |
| **IS** | 0.94 | **1.37*** | **1.75** | 1.01 | 1.07 | **1.63** |
| **IT** | **2.18*** | **1.33** | **1.76*** | 1.60 | **1.40** | 1.54 |
| **KZ** | **1.53** | 1.10 | **3.30** | 1.26 | 1.25 | 1.52 |
| **LT** | 0.82 | 1.11 | **1.66** | 1.27 | 1.11 | 1.39 |
| **LU** | 1.23 | 0.97 | **2.05*** | 1.59 | **1.80*** | **2.32*** |
| **LV** | 0.80 | **1.40** | **1.54** | 1.05 | 1.35 | 0.97 |
| **MD** | 0.93 | 1.16 | **1.71** | 0.95 | 0.90 | 1.06 |
| **MT** | 0.56 | 0.89 | 0.51 | 1.19 | 1.05 | 0.74 |
| **NL** | 0.98 | 0.97 | 1.28 | 1.29 | 1.16 | 0.57 |
| **NO** | 0.91 | 1.06 | **1.60*** | 1.36 | 0.99 | 1.47 |
| **PL** | 0.79 | 1.07 | **1.88*** | 0.73 | 1.26 | 1.20 |
| **PT** | 0.93 | 1.07 | **1.61** | 1.08 | 1.20 | 1.10 |
| **RO** | 1.49 | 1.09 | **1.67*** | 1.08 | 0.97 | 1.37 |
| **RU** | 0.71 | 0.97 | 1.34 | 1.11 | 1.04 | 1.63 |
| **SE** | 1.13 | 1.23 | 1.48 | 0.91 | 1.25 | 1.46 |
| **SI** | 0.93 | 1.04 | 1.45 | 1.01 | 1.08 | 1.09 |
| **UA** | 0.88 | 1.21 | **1.99*** | 1.52 | 1.34 | **1.81** |
| **TR** | 0.97 | 1.04 | **1.56** | 0.97 | 0.95 | 1.03 |
| In bold if p<0.05; * if p<0.01 | | | | | | |

Abbreviations. A.L., Albania; AM, Armenia; AT, Austria; AZ, Azerbaijan; BE-VLG, Belgium (French); BE-WAL, Belgium (Flemish), CA, Canada; C.H., Switzerland; CZ, Czech Republic; DE, Germany; DK, Denmark; E.E. Estonia; ES, Spain; FI, Finland; FR, France; GE, Georgia, GR, Greece; H.R., Croatia; H.U., Hungary; IL, Israel; I.S., Iceland; I.T., Italy; K.Z., Kazakhstan; L.T., Lithuania; LU, Luxemburg; LV, Latvia; M.D. Republic of Moldova; MT, Malta; N.L., Netherlands; NO, Norway; PL, Poland; PT, Portugal; R.O., Romania; R.U., Russia; SE, Sweden; S.I., Slovenia; T.R., Turkey; U.A., Ukraine.
